# Cordyceps Polysaccharides Attenuate Post-Ischemic Inflammatory Damage in a Rat Stroke Model with Associated Modulation of the mtDNA/NLRP3 Inflammasome-Related Pyroptosis Pathway

**DOI:** 10.3390/nu18162591

**Published:** 2026-08-07

**Authors:** Yifan Chen, Huizhang Wang, Cong Gai, Xia Li, Yutong Li, Yihui Ma, Huiming Qi, Changhua Shi, Yibo Tang

**Affiliations:** School of Traditional Chinese Medicine, Beijing University of Chinese Medicine, Beijing 102488, China

**Keywords:** Cordyceps polysaccharides, ischemic stroke, CMPK2, NLRP3 inflammasome, pyroptosis

## Abstract

Background: Cordyceps polysaccharides (CSP) have shown neuroprotective potential in models of ischemic stroke, but the underlying mechanisms remain to be clarified. Methods: In this study, the effects of CSP were evaluated in MCAO rats and OGD-exposed BV-2 cells. Results: CSP significantly attenuated ischemic injury and inflammatory responses in both in vivo and in vitro models. Mechanistically, CSP decreased CMPK2 expression, increased TFAM levels, and reduced 8-OHdG expression, suggesting attenuation of oxidative DNA damage and mitochondrial DNA-associated stress. EdU staining further showed that OGD-induced DNA synthesis-related signals were predominantly extranuclear, supporting the possibility of mtDNA-associated alterations under ischemia-like conditions. Moreover, CSP suppressed the upregulation of NLRP3, Caspase-1, *N*-GSDMD, IL-1β, and IL-18, and reduced LDH release following OGD exposure, suggesting inhibition of inflammasome-associated pyroptotic signaling. Conclusions: These findings suggest that the neuroprotective effects of CSP in ischemic stroke models may be related to the modulation of CMPK2/mtDNA/NLRP3 inflammasome pathway and reduced pyroptosis.

## 1. Introduction

Stroke is among the most prevalent cerebrovascular disorders and remains the second leading cause of death worldwide, accounting for approximately 6 million deaths annually and 11.6% of all-cause mortality [1]. Based on its underlying pathogenesis, stroke is broadly categorized into hemorrhagic stroke (HS) and ischemic stroke (IS), of which IS constitutes nearly 86% of all cases [2]. At present, tissue-type plasminogen activator (tPA) remains one of the few approved pharmacological therapies for acute ischemic stroke [3]. While demonstrating significant thrombolytic effects, tPA may also induce reperfusion injury, leading to massive leukocyte infiltration into ischemic tissues and exacerbating inflammatory damage [4]. These limitations underscore the urgent need to identify novel pharmacological strategies for IS. In this context, traditional Chinese medicines and their bioactive constituents have attracted increasing attention as potential therapeutic candidates.

Cordyceps polysaccharides (CSP) are among the most abundant and extensively investigated bioactive constituents of Cordyceps sinensis [5]. Increasing evidence suggests that these polysaccharides exert beneficial effects in models of ischemic stroke by attenuating neuroinflammation, oxidative stress, and apoptosis following cerebral ischemia [6]. Our previous studies likewise demonstrated that Cordyceps polysaccharides confer both preventive and protective effects in experimental models of ischemic stroke [7]; at least in part through suppression of microglial activation and subsequent inflammatory responses. However, the precise molecular mechanisms underlying these neuroprotective effects remain to be fully elucidated. To provide a pharmacological reference for the effects of CSP in the present study, Naoxintong capsule (NXT) was used as a positive control. NXT is a compound traditional Chinese medicine formula composed of multiple plant- and animal-derived medicinal materials, such as *Astragalus membranaceus* and *Salvia miltiorrhiza*. Its reported bioactive constituents mainly include saponins and phenolic acids. NXT has been extensively investigated in cerebrovascular disease models, and previous studies have shown that it can reduce cerebral infarction severity and improve neurological function in Middle Cerebral Artery Occlusion (MCAO) rats [8].

With the deepening understanding of IS pathogenesis, considerable progress has been made in the development of diagnostic and therapeutic approaches. Nevertheless, the pathological basis of IS remains highly complex, involving a series of interconnected events, involving excitotoxicity [9], oxidative stress [10], inflammatory responses [11], and apoptosis [12]. These intricate pathological processes interconnect, ultimately leading to structural damage and persistent functional impairment of the central nervous system [13]. Among the multiple mechanisms implicated in ischemic stroke, mitochondrial dysfunction has emerged as a pivotal contributor [14]. As the principal organelles responsible for cellular energy metabolism, mitochondria are critically involved in several major pathological processes associated with stroke, including oxidative stress [15], inflammation [16], and apoptosis [17]. Mitochondria also harbor their own genome, mitochondrial DNA (mtDNA), a closed circular double-stranded DNA molecule essential for mitochondrial homeostasis and function [18]. Importantly, under conditions of cellular stress or injury, mtDNA can act as a damage-associated molecular pattern (DAMP), thereby initiating and amplifying inflammatory signaling [19]. Cytidine/uridine monophosphate kinase 2 (CMPK2) is localized in mitochondria and acts as a rate-limiting enzyme for maintaining intracellular DNTPs levels, being essential for mitochondrial DNA synthesis.

Recent studies have highlighted cytidine/uridine monophosphate kinase 2 (CMPK2) as a key regulator of mitochondrial nucleotide metabolism and mtDNA synthesis. CMPK2 is a mitochondria-localized enzyme that functions as a rate-limiting factor in maintaining intracellular UTP/CTP pools and is required for inflammation-associated de novo mtDNA synthesis [20]. Under physiological conditions, transcription factor A mitochondrial (TFAM), packages mtDNA into nucleoids, thereby limiting its exposure and reducing mtROS-mediated damage [21]. Under stimulation such as ischemia or LPS, however, CMPK2 expression is markedly upregulated, promoting de novo mtDNA synthesis by supplying the deoxyribonucleotide substrates required for mitochondrial genome replication. Meanwhile, neuronal injury and death drive excessive microglial activation, particularly in ischemic and hypoxic regions such as the penumbra, where mitochondrial ROS production is substantially increased. Newly synthesized mtDNA is therefore highly susceptible to oxidative modification, resulting in the accumulation of oxidized mtDNA (Ox-mtDNA) [22]. In parallel, Ca^2+^ transfers from the endoplasmic reticulum into mitochondria through the mitochondrial calcium uniporter (MCU) complex facilitates opening of the mitochondrial permeability transition pore (mPTP). Ox-mtDNA may then be processed by flap endonuclease 1 (FEN1) into smaller DNA fragments [23], which are released into the cytoplasm through mPTP and voltage-dependent anion channel (VDAC)-associated pathways, where they associate with and activate the NLRP3 inflammasome. Subsequent activation of Caspase-1 promotes cleavage of gasdermin D (GSDMD), generating the pore-forming *N*-terminal fragment and enabling the release of mature pro-inflammatory cytokines, including IL-1β and IL-18. This cascade amplifies neuroinflammation and further aggravates ischemic brain injury.

Collectively, our previous studies have shown that CSP has neuroprotective and anti-inflammatory effects in models of ischemic stroke [7], whereas the signaling pathways associated with these effects remain unclear. Because mitochondrial dysfunction, mtDNA dysregulation, and NLRP3 inflammasome activation are critically involved in post-ischemic neuroinflammation and neuronal injury, we focused on the CMPK2/TFAM/mtDNA/NLRP3 inflammasome-related pathway as a candidate mechanism. Therefore, the present study aimed to determine whether CSP attenuates ischemic brain injury in vivo and in vitro and whether this protection is accompanied by changes in CMPK2 expression, TFAM-associated mtDNA maintenance, mtDNA-related inflammatory signaling, and NLRP3 inflammasome-mediated pyroptosis. The proposed mechanism is summarized in the graphical abstract.

## 2. Materials and Methods

### 2.1. Preparation of CSP and NXT for In Vivo Experiments

Cordyceps polysaccharides was acquired from Shanghai Yuanye BioTechnology Co., Ltd. (Shanghai, China, Cat. S27812-100g). Naoxintong Capsule was acquired from Buchang Pharmaceutical (Shanxi, Xi’an, China, Cat.220025001). The rat dose of CSP used in this study was 1 g/kg by gavage, which was selected based on our preliminary dose-exploration experiments showing a stable protective effect in the ischemic stroke model [7,24]. 10 g of CSP powder and 10 g of Naoxintong (NXT) powder were separately dissolved in distilled water and diluted to a final volume of 200 mL, yielding stock solutions containing 0.05 g/mL CSP and 0.05 g/mL NXT, respectively. The prepared solutions were stored at −20 °C and warmed to 37 °C before use.

### 2.2. Middle Cerebral Artery Occlusion (MCAO) Model Preparation

Male Sprague–Dawley rats (180–220 g) were purchased from Sibeifu Animal Technology Co., Ltd. (China, Beijing, SYXK 2019-0010). The experimental protocol was reviewed and approved by the Animal Experiment Ethics Committee of Beijing University of Chinese Medicine (BUCM20250910-009). The rats were acclimatized for 7 days at the Animal Experimental Center of Beijing University of Chinese Medicine. During the acclimatization period, they were allowed free access to food and water. The temperature was maintained at 20 ± 2 °C, the humidity was kept at 55–60%, and the animals were housed under natural light conditions. Feed was replaced regularly. The rats were divided into four groups using a random number table: the Sham group, the MCAO group, the MCAO + CSP group (1 g/kg, i.g.), and the MCAO + NXT group (1 g/kg, i.g.) [7]. After 7 days of acclimatization, the NXT group and the CSP group received NXT and CSP for five consecutive days, while the Sham and MCAO groups received equal volumes of physiological saline. 1 h prior to modeling on day 6, the drug was administered again, followed by anesthesia induction and maintenance using a gas anesthesia machine containing isoflurane (3% for induction, 2.5% for maintenance).

Consistent with our previous studies, using the filament technique to establish a right MCAO model [25]: The right common carotid artery (CCA), external carotid artery (ECA), and internal carotid artery (ICA) were dissected, a filament was inserted through the CCA incision and advanced along the ICA to the origin of the middle cerebral artery (MCA); The sham group only isolated blood vessels without performing embolization, excluding the influence of vascular isolation itself on cerebral infarction. The MCAO, CSP, and NXT groups underwent MCAO modeling. In the in vivo experiments, the MCAO + CSP and MCAO + NXT groups refer to rats subjected to MCAO modeling combined with CSP or NXT administration respectively.

Postoperatively, thermal protection and infection prevention were ensured. After awakening, rats were assessed using the Longa scoring system. A score of ≥1 confirmed the successful establishment of the MCAO model, while rats scoring 0 or 4 were excluded from the study. In each group, rats received oral administration of the drug or saline at the same time daily after model creation. Samples were collected 72 h after surgery. A total of 63 rats were used: Sham, MCAO, and MCAO + CSP groups each included 18 rats, while the MCAO + NXT group included 9 rats. Six rats from each group were used for laser speckle contrast imaging and TTC staining. In the Sham, MCAO, and MCAO + CSP groups, the remaining 12 rats per group were used for Western blot analysis and paraformaldehyde fixation, with six rats allocated to each experiment. In the MCAO + NXT group, the remaining three rats were perfused and fixed for H&E and Nissl staining. NXT was used as a positive control, and mechanistic analyses were therefore focused on the Sham, MCAO, and MCAO + CSP groups.

### 2.3. Corner Experiment

The corner test was performed to assess sensorimotor asymmetry after ischemic stroke. Two wooden boards were fixed at a 30° angle to form a turning device. Rats were placed at the opening of the angle, allowing them to freely enter the interior and turn outward, with the number of left or right turns recorded. Each rat underwent 10 consecutive tests, with intervals of no less than 30 s, and the proportion of turns toward the affected side was calculated relative to the total number of turns to assess the degree of post-ischemic sensory-motor hemilateralization.

### 2.4. Neurological Function Scores (mNSS)

The mNSS test was used to evaluate neurological deficits after MCAO. After awakening from MCAO modeling and 72 h postoperatively, the neurological impairment severity in each group of rats was assessed using the Modified Neurological Severity Score (mNSS) Scale. The scoring primarily evaluated motor function, sensory function, balance ability, and reflex activity, and was independently performed by two laboratory personnel unaware of the group assignments. The specific evaluation criteria and scoring standards for the mNSS scales are detailed in Supplementary Table S1 [26].

### 2.5. Laser Speckle

Laser speckle contrast imaging was performed to evaluate cortical cerebral blood flow after MCAO. The rats were anesthetized with 2.5% isoflurane via inhalation and immobilized in a prone position. The scalp was incised to expose the skull, which was continuously kept moist with normal saline. The laser speckle blood flow imaging probe was positioned approximately 15 cm above the skull, and cortical blood flow was continuously monitored in the ischemic and contralateral hemispheres was continuously monitored after clear images were obtained. Bilateral cerebral blood flow values were analyzed using the Moor FLPI-2 V4.0 software, and the cerebral blood flow ratio between the affected and contralateral regions was calculated. The calculation formula was ipsilateral blood flow/contralateral blood flow × 100%.

### 2.6. 2,3,5-Triphenyltetrazolium Chloride Staining (TTC)

TTC staining was performed to assess cerebral infarct volume. After brain harvesting, the cerebellum, brainstem, and olfactory bulb were carefully removed. After freezing at −20 °C for approximately 10 min, the brain tissue was uniformly sectioned into five slices approximately 2 mm thick along the coronal plane. Brain slices were incubated in 2% TTC solution at 37 °C for 20 min protected from light, with gentle stirring to ensure uniform staining, followed by fixation with 4% paraformaldehyde. Normal brain tissue regions appeared red, while infarcted areas showed white staining. Images were captured and results were recorded. The percentage of infarction was calculated as the infarct area divided by the area of the whole sections, multiplied by 100%.

### 2.7. Brain Index Measurement

The brain index was calculated to evaluate brain swelling after ischemic injury. The whole brain was collected, and surface blood and moisture were gently removed before weighing, and the blood and moisture on the surface of the brain tissue were aspirated. The wet weight of the entire brain was then measured, and the body weight of the rats was recorded simultaneously. The calculation formula is Brain Index = Brain Wet Weight/Body Weight × 100%.

### 2.8. Brain Water Content Analysis

Brain water content was measured to assess cerebral edema. The water content of brain tissue was determined using the dry-wet weight method. Fresh brain tissue was weighed for its wet weight, then dried at 60 °C in a constant-temperature oven until a constant dry weight was obtained, after which the dry weight was measured. The formula for calculation is Water content of brain tissue = (wet weight-dry weight)/wet weight × 100%.

### 2.9. Hematoxylin and Eosin Staining (H&E)

H&E staining was performed to evaluate histopathological changes in the ischemic penumbra. Rats were transcardially perfused with phosphate-buffered saline followed by 4% paraformaldehyde under anesthesia. Brain tissue samples were collected and then immersed in 4% paraformaldehyde for 48 h. The brain tissues were further processed by paraffin embedding, paraffin sectioning (with a thickness of 4 μm), dewaxing, and staining with hematoxylin for 3 min and eosin for 1 min. Following dehydration, transparency treatment, and sealing, pathological changes in the ischemic penumbra were compared among different groups using a light microscope.

### 2.10. Nissl Staining

Nissl staining was performed on paraffin-embedded rat brain sections. For MCAO rats, sections containing the ischemic penumbra region of the ipsilateral hemisphere were used, while the corresponding brain region was selected from Sham rats. The paraffin section preparation method was identical to that described above. After dewaxing with xylene, the sections were successively rehydrated in graded ethanol solutions of 100%, 95%, 80%, and 70%. Staining was performed using Nissl staining solution in a 37 °C oven for 20 min, followed by washing with distilled water, differentiation with 95% ethanol for 1 min, dehydration through graded ethanol, clearing with xylene, and mounting. The morphology and distribution of neuronal Nissl bodies in the ischemic penumbra or corresponding brain region were observed under an optical microscope. Identical brain regions were selected for observation and image acquisition in each group for comparative analysis.

### 2.11. Immunohistochemistry (IHC)

Immunohistochemistry was performed to detect the expression and distribution of inflammation- and oxidative DNA damage-related markers in the ischemic penumbra. The paraffin section preparation method was identical to that described above. After dewaxing with xylene, the paraffin sections were successively rehydrated in gradient solutions of 100%, 95%, 80%, and 70% ethanol, followed by rinsing with distilled water. Microwave-induced antigen retrieval was then performed using EDTA buffer antigen retrieval solution. After natural cooling to room temperature, the sections were washed with PBS. The sections were blocked for 30 min using blocking solution from the Proteintech immunohistochemistry kit PK10006. After washing three times with TBS solution for 5 min each, the sections were incubated overnight at 4 °C with primary antibodies against Iba1 (1:300, Abcam, ab178846, Cambridge, UK), NLRP3 (1:300, ABMART, P60622R3, Shanghai, China), and 8-OHdG (1:300, ABMART, PC1278, Shanghai, China). The next day, the sections were washed three times with TBS solution for 5 min each, followed by addition of the corresponding secondary antibodies from the kit and incubation at room temperature for 1 h. DAB staining was performed for 3 min, after which the sections were immersed in pure water to terminate the staining reaction. Nuclei were counterstained with hematoxylin for 5 min, followed by dehydration with successive alcohol layers, cleared in xylene, and sealing with neutral resin. The sections were then observed and images were acquired under an optical microscope. The same brain regions were selected in each group using Image J for observation and image acquisition, and comparative analysis was performed using GraphPad Prism 10.1.2. For each rat, one paraffin section from the ischemic penumbra or corresponding brain region was selected, and one microscopic field was randomly chosen for quantitative analysis. Each section was obtained from an independent rat, with *n* = 6 rats per group.

### 2.12. Preparation of CSP-Containing Serum for In Vitro Experiments

Ten healthy male Sprague–Dawley rats weighing approximately 300 g were used for the preparation of CSP-containing serum. After 3 days of acclimatization, the rats were orally administered CSP at a dose of 2 g/kg three times daily for five consecutive days. At 2 h after the final administration, blood was collected under isoflurane inhalation anesthesia and allowed to stand undisturbed for 2 h at room temperature. The samples were then centrifuged to isolate the serum. Approximately 70 mL of CSP-containing serum was obtained from the 10 rats.

The collected serum was heat-inactivated at 56 °C for 30 min to inactivate complement components. Because the serum was subsequently used for direct treatment of cultured cells, it was filtered through a 0.22 μm sterile membrane under aseptic conditions to reduce the risk of microbial contamination, and then stored at −80 °C until use.

Blank serum was prepared from rats of the same batch that received an equal volume of physiological saline by oral gavage following the same procedure. Approximately 70 mL of blank serum was obtained from 10 rats in the blank control group. For cell experiments, CSP-containing serum was diluted with blank serum so that the final total serum concentration in the culture medium was maintained at 20% in all treatment groups. The final concentrations of CSP-containing serum were 1.25%, 2.5%, 5%, 10%, 15%, and 20%, with the remaining serum proportion supplemented by blank serum. For example, the 1.25% CSP-containing serum group contained 1.25% CSP-containing serum and 18.75% blank serum, while the 20% CSP-containing serum group contained 20% CSP-containing serum without additional blank serum.

After treatment, BV-2 cells and culture supernatants from each group were collected for subsequent in vitro analyses, including assessment of cell viability, detection of inflammatory responses, and measurement of inflammation-, mitochondrial dysfunction-, mtDNA-, and pyroptosis-related markers.

### 2.13. Cell Culture and Oxygen-Glucose Deprivation (OGD) Model Preparation

Mouse microglial cells BV-2 (Wuhan Shangen Biotechnology Co., Ltd., Wuhan, China, 202300515shu-03) were used for in vitro experiments. The culture was performed using BV-2-specific complete medium, with 5 mL of the medium added to T25 flasks.

Logarithmically growing BV-2 cells were trypsin-digested to prepare a single-cell suspension. After adjusting the cell density, the suspension was uniformly seeded onto corresponding culture plates and incubated under routine conditions at 37 °C with 5% CO_2_. The study was divided into 3 groups: Control group (CON), OGD group, and OGD + CSP-containing serum group. In the in vitro experiments, the OGD + CSP-containing serum group refers to BV-2 cells pretreated with CSP-containing serum for 24 h before OGD modeling, rather than cells treated with CSP-containing serum alone. Cells were seeded in culture plates according to the aforementioned grouping. When cells reached a specific growth stage, the CON group continued to be cultured in complete medium, whereas the OGD group and CSP group were switched to glucose-free medium and placed in an hypoxic three-gas incubator (containing 5% CO_2_, 1% O_2_, and 94% N_2_) at 37 °C. Culturing for 6 h established the OGD model, which simulates cerebral ischemia through glucose and oxygen deprivation [27].

### 2.14. Cell Viability Assay Using CCK-8

Cell viability was assessed using the CCK-8 assay. BV-2 cells in the logarithmic growth phase were harvested, digested with trypsin to prepare a single-cell suspension, and resuspended at a density of 5 × 10^3^ to 1 × 10^4^ cells per well. The cells were seeded in 96-well plates, with 100 μL of complete culture medium added per well, and incubated under routine conditions at 37 °C with 5% CO_2_ for 24 h until the cells achieved optimal adhesion. Experimental groups included: CON group, OGD group, and CSP serum groups with varying concentrations (1.25%, 2.5%, 5%, 10%, 15% and 20%). Prior to modeling, the drug-treated groups were pre-treated with the corresponding gradient concentrations of CSP-containing serum culture medium 24 h before modeling; the CON and OGD groups received an equal volume of complete culture medium followed by OGD modeling. After modeling, 10% CCK-8 reagent was added per well, gently mixed, and incubated at 37 °C in the dark for 1 h. Absorbance was measured at 450 nm using a microplate reader. Based on the CCK-8 results, 5% CSP-containing serum was selected for subsequent in vitro experiments.

### 2.15. EdU Staining Experiment

EdU staining was performed as a complementary assay to assess DNA synthesis-related changes in BV-2 cells under OGD conditions and to provide supportive evidence for the effects of CSP-containing serum on cellular responses to ischemic-like injury. The BV-2 cells were seeded in two 6-well plates and divided into the CON group, OGD group, and CSP group according to the experimental design, with six replicates per group (*n* = 6). After the cells had attached and stabilized, the OGD and CSP groups underwent OGD modeling. Subsequently, 5-ethynyl-2′-deoxyuridine (EdU) working solution was added to each well to achieve a final concentration of 10 μM, and incubation continued for 2 h. The culture medium was discarded, followed by 15 min fixation at room temperature with 4% paraformaldehyde, three washes, and 10–15 min of permeabilization at room temperature with 0.3% Triton X-100. Then, 500 μL of Click reaction solution was added to each well, and incubation was performed at room temperature for 30 min. After washing, nuclei were counterstained with Hoechst 33342 for 10 min. Finally, images were captured using a fluorescence microscope, and the percentage of EdU-positive cells and total EdU fluorescence intensity were quantified. Because EdU incorporation reflects overall DNA synthesis-associated activity and may include a contribution from mtDNA synthesis, total EdU fluorescence intensity was used only as a supportive indicator and was interpreted together with other mitochondrial and inflammatory markers.

### 2.16. Enzyme-Linked Immunosorbent Assay (ELISA)

ELISA was performed to quantify 8-OHdG levels in BV-2 cells after OGD and CSP-containing serum treatment. The cells were divided into the CON group, OGD group, and OGD+CSP group, with six replicates per group. After corresponding treatments and completion of OGD modeling, the cells were collected and total protein was extracted. Protein concentrations were determined using the BCA method, and sample concentrations were adjusted to be consistent. Detection was performed according to the instructions of the 8-OHdG ELISA kit: 50 μL of standard solution and the sample were added to the standard wells and sample wells, respectively. Immediately, 50 μL of biotinylated antibody working solution was added per well, followed by incubation at 37 °C for 45 min. After three washes, 100 μL of HRP conjugate working solution was added per well, and incubation continued at 37 °C for 30 min. Subsequently, after five additional washes, 90 μL of TMB substrate solution was added per well, and the plate was incubated at 37 °C protected from light for approximately 15 min. The reaction was terminated by adding 50 μL of termination buffer. Finally, the absorbance of each well was measured at a wavelength of 450 nm, and the 8-OHdG levels in each group were calculated based on the standard curve.

### 2.17. Lactate Dehydrogenase Release Assay (LDH)

LDH release was measured to assess cell membrane damage and cytotoxicity. BV-2 cells in logarithmic growth phase were inoculated into 96-well plates and divided into the CON group, OGD group, and CSP group. After respective treatments and completion of OGD modeling, the cell culture supernatants were collected. The LDH working solution was prepared by mixing 91 μL of LDH release assay buffer with 9 μL of chromogen solution for each reaction well. Samples were added to 96-well plates and supplemented with LDH release assay buffer to a final volume of 100 μL. Then, 100 μL of LDH detection working solution was added to each well. The plate was mixed gently and incubated at 37 °C in the dark for 10–30 min. After the reaction, 20 μL of stop solution was added to each well, and absorbance was measured immediately at 450 nm.

### 2.18. Western Blot (WB)

Western blotting was performed to detect the expression of inflammation-, mitochondrial dysfunction-, and pyroptosis-related proteins in brain tissue and BV-2 cells. Proteins were extracted from ischemic penumbra brain tissue and BV-2 cells using RIPA lysis buffer supplemented with protease and phosphatase inhibitors. Approximately 50 mg of brain tissue was homogenized in 500 μL of lysis buffer. Cell samples were lysed directly in RIPA lysis buffer. Lysates were incubated on ice for 30 min and then centrifuged at 12,000 rpm for 15 min at 4 °C. Protein concentrations were determined using a BCA assay.

The WB was performed using a 10% gel for separation, followed by 30 min of concentration at 80 V and then 160 V for separation. After electrophoresis, the membrane was transferred to a PVDF membrane under wet conditions, activated with ethanol for 30 s, and transferred at 400 mA for 30 min under ice bath conditions. The membrane was blocked with 5% non-fat milk at room temperature for 2 h. Primary antibodies were added: CMPK2 (1:2000, Abcam, ab194567, Cambridge, UK), TFAM (1:2000, ZhongHe, ZHS1147, Beijing, China), IL-1β (1:2000, ZhongHe, ZHS5008, Beijing, China), Caspase-1 (1:1000, ABMART, P79884R2S, Shanghai, China), *N*-GSDMD (1:2000, ZhongHe, ZHS5040, Beijing, China), IL-18 (1:2000, Proteintech, 10663-1-AP, Wuhan, China), NLRP3 (1:2000, ABMART, P60622R3, Shanghai, China), and β-Actin (1:5000, Lablead, A1101, Beijing, China). Incubation was carried out overnight at 4 °C. After washing with TBST, the secondary antibody was added and incubated at room temperature for 1 h. The membrane was washed again, and protein bands were visualized using NcmECL Ultra (NCM Biotech, P10100). The relative expression levels of CMPK2, TFAM, IL-1β, Caspase-1, *N*-GSDMD, IL-18, and NLRP3 were normalized to β-Actin.

### 2.19. Statistical Analysis

All data are presented as mean ± SD. The sample size for each experiment is indicated in the corresponding figure legends. Normality was assessed using the Shapiro–Wilk test. For normally distributed data, differences among multiple groups were analyzed by one-way analysis of variance ANOVA. When multiple treatment groups were compared with a single control group, Dunnett’s multiple comparisons test was used for post hoc analysis, and adjusted *p* values and 95% confidence intervals were reported. For data that did not meet the normality assumption or showed borderline deviation from normality, the Kruskal–Wallis test followed by Dunn’s multiple comparisons test was used. For in vivo experiments, comparisons were mainly performed between the MCAO group and the Sham group, and between the treatment groups and the MCAO group. For in vitro experiments, comparisons were mainly performed between the OGD group and the CON group, and between the treatment groups and the OGD group. All statistical tests were two-sided, and *p* < 0.05 was considered statistically significant. Statistical analyses were performed using GraphPad Prism software.

## 3. Results

### 3.1. CSP Improved Neurological Function and Attenuated Histopathological Damage Following Ischemic Brain Injury

**Figure 1:**
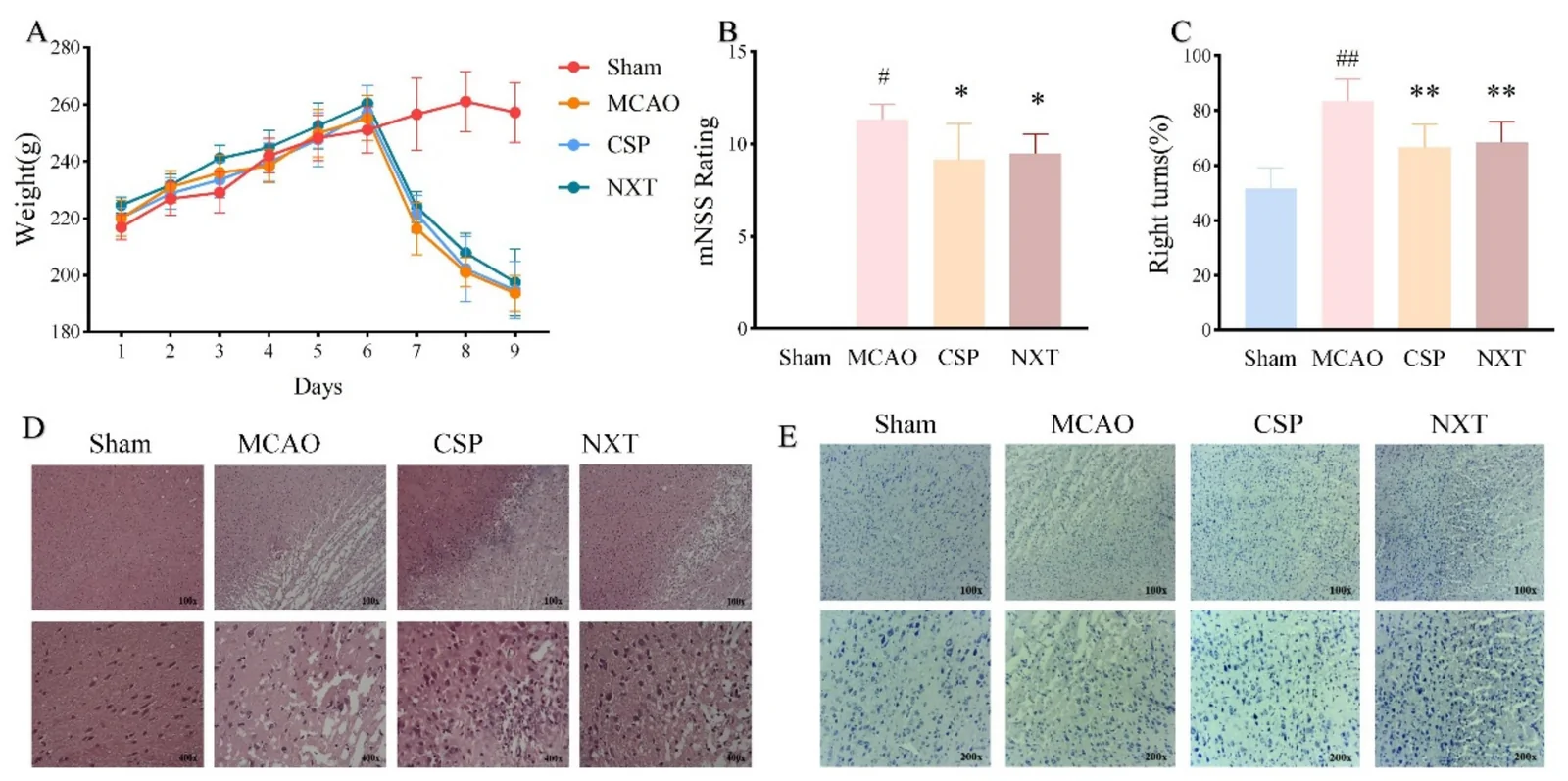
Body weight, mNSS, corner turn test (*n* = 6), H&E staining and Nissl staining (*n* = 3). (**A**) Changes in body weight. (**B**) mNSS scores. (**C**) Corner turn test. (**D**) H&E staining result image. Magnification: 100× and 400×. (**E**) Nissl staining result image. Magnification: 100× and 200×. Data are expressed as the mean ± SD. ^#^ *p* < 0.05, ^##^ *p* < 0.01 vs. the Sham group and * *p* < 0.05, ** *p* < 0.01 vs. the MCAO group. In this figure and subsequent in vivo figures, “CSP” denotes MCAO rats treated with CSP, and “NXT” denotes MCAO rats treated with NXT.

In-depth pharmacodynamic studies were conducted to confirm the protective effect of CSP in the MCAO model before mechanistic investigation. Pharmacodynamic experiments were repeated using independent animal cohorts, following our previously established protocol. All groups exhibited stable weight gain during the feeding and pre-treatment phases. NXT was used as a positive control to validate the responsiveness of the MCAO model and to benchmark the neuroprotective effect of CSP. The Sham group showed no significant weight change before or after modeling, whereas the MCAO group, CSP group, and NXT group all demonstrated significant weight loss on day 3 post-modeling (Figure 1A). The MCAO group had significantly higher mNSS scores than the Sham group. Conversely, the CSP group and NXT group demonstrated significantly lower mNSS scores compared to the MCAO group, indicating significant improvement in neurobehavioral function (Figure 1B). The Sham group exhibited approximately equal left and right turn frequencies, while the MCAO group showed a marked increase in rightward turn frequency. The CSP group and NXT group exhibited significantly lower rightward turn frequencies compared to the MCAO group (Figure 1C).

H&E staining results showed no abnormalities in the Sham group. In the ischemic penumbra region of the MCAO group, most cortical cells exhibited atrophy, disordered arrangement, and morphological deformation, with extensive neuronal necrosis characterized by nuclear shrinkage. Compared to the MCAO group, both the CSP group and NXT group demonstrated reduced structural damage within the ischemic penumbra (Figure 1D). Nissl staining results showed dense neurons and Nissl bodies in the Sham group. The MCAO group exhibited neuronal disorganization and deformation, along with a significant reduction in Nissl body count. In contrast, the CSP group and NXT group displayed more orderly microstructural features and increased Nissl body quantity compared to the MCAO group (Figure 1E).

### 3.2. CSP Reduced Cerebral Infarct Volume and Improved Cerebral Blood Flow

**Figure 2:**
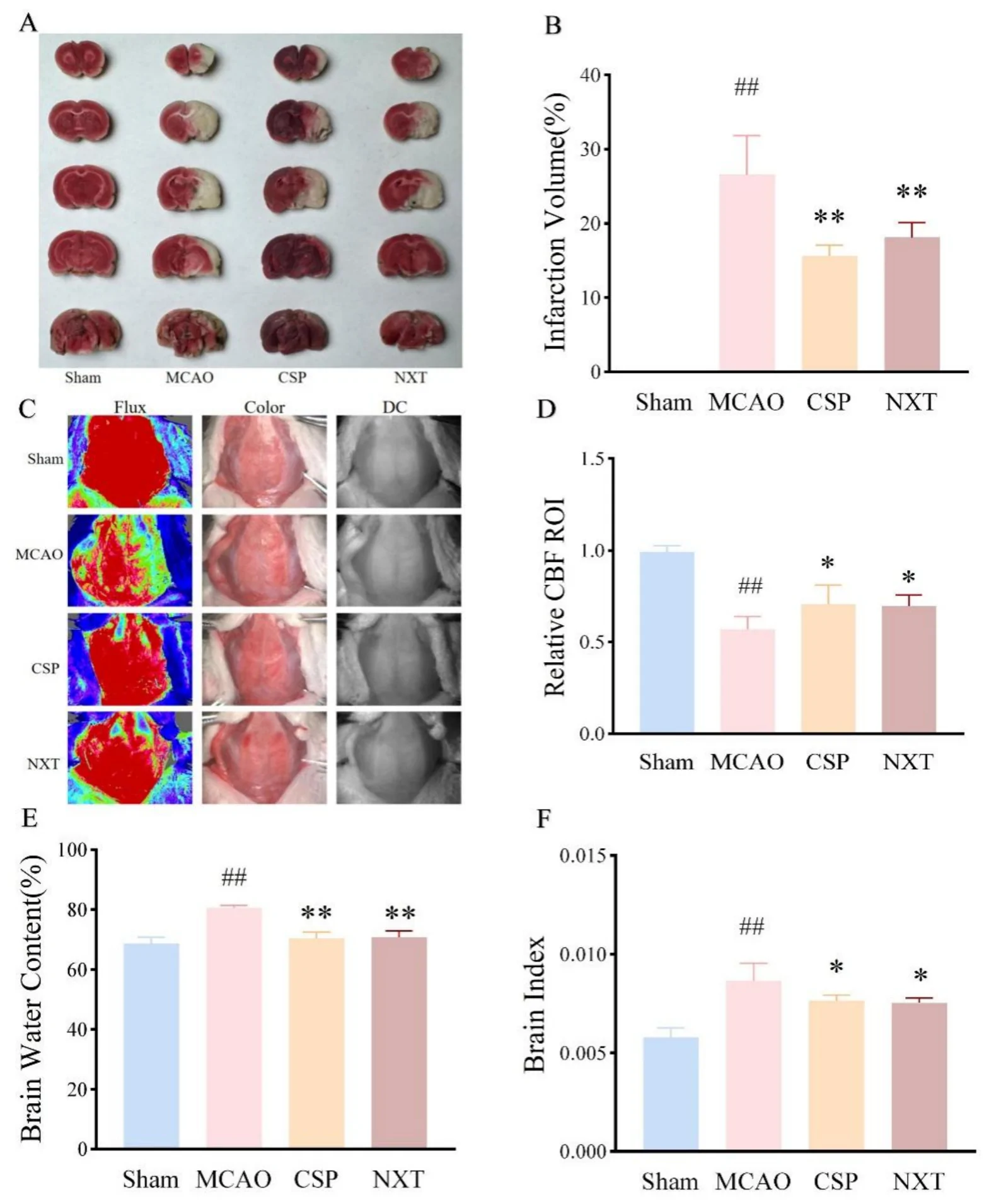
TTC staining and Laser speckle contrast imaging (*n* = 6). (**A**) Representative images of TTC. (**B**) Quantitative diagram of TTC staining. (**C**) Laser speckle Flux/Color/DC demonstration image. (**D**) Cerebral blood flow measurements. (**E**) Brain tissue water content in rats. (**F**) Brain index of rats. Data are expressed as the mean ± SD. ^##^ *p* < 0.01 vs. the Sham group and * *p* < 0.05, ** *p* < 0.01 vs. the MCAO group.

Compared with the Sham group, the MCAO group exhibited a significantly increased cerebral infarct volume and a marked reduction in ipsilateral cerebral blood flow. Compared with the MCAO group, both the CSP and NXT groups showed a significantly reduced cerebral infarct volume and markedly increased ipsilateral cerebral blood flow (Figure 2A–D). Brain edema and brain index were significantly increased in the MCAO group compared with the Sham group. In contrast, CSP and NXT treatment significantly reduced brain edema and brain index compared with the MCAO group (Figure 2E,F).

### 3.3. CSP Improved Ischemic Injury in Rats by Ameliorating Mitochondrial Dysfunction in Microglia Through CMPK2

**Figure 3:**
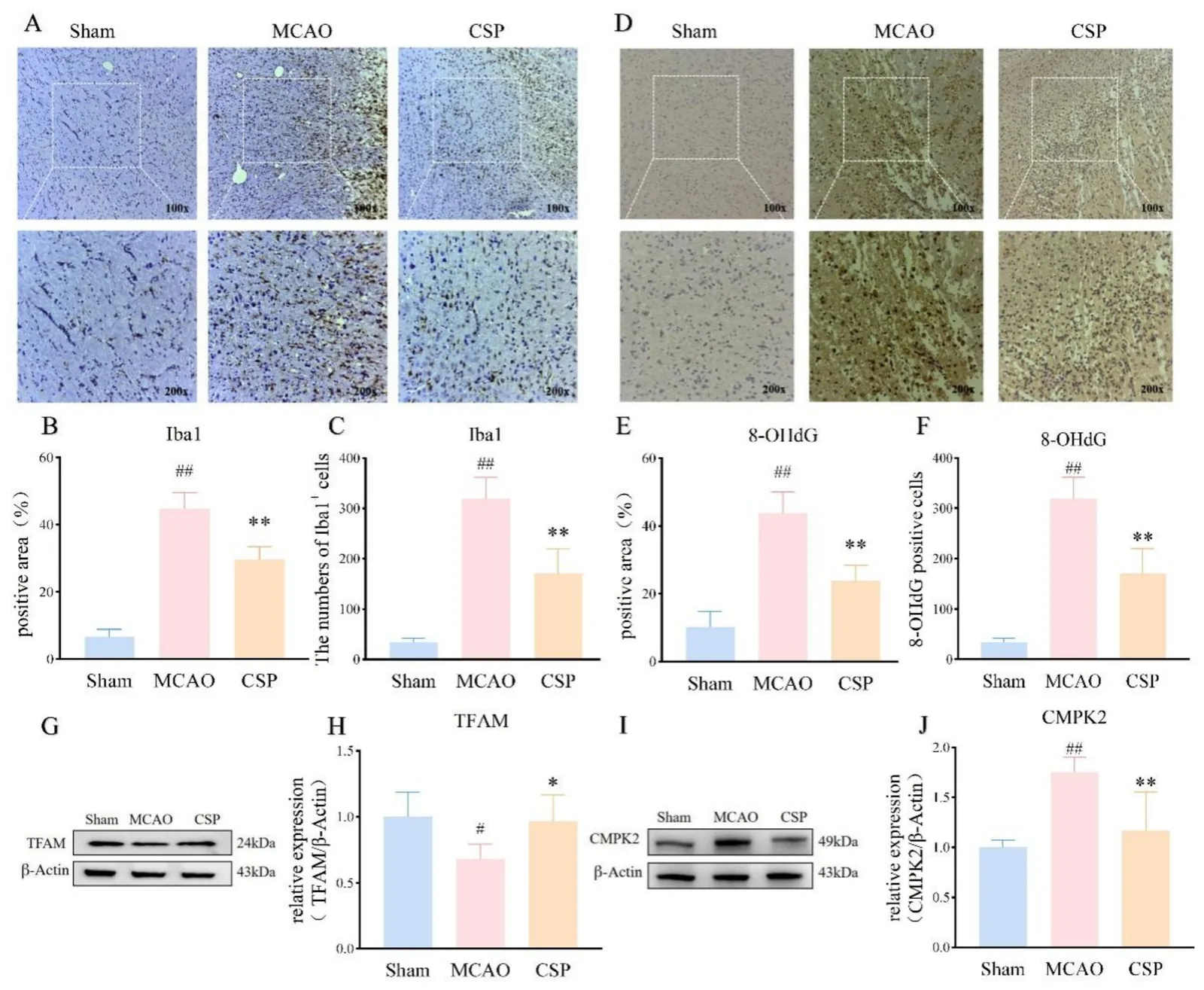
Microglial Activation, Oxidative Damage and Mitochondrial Protein Changes in Ischemic Penumbra of MCAO Rats (*n* = 6). (**A**) IHC images showcasing microglia in the ischemic penumbra. (**B**) Calculation of Iba1 positive area. (**C**) Quantitative diagram of Iba1 positive cells. (**D**) IHC images demonstrate the expression of 8-OHdG in the ischemic penumbra. (**E**) Calculation of 8-OHdG positive area. (**F**) Quantitative diagram of 8-OHdG positive cells. (**G**) Representative pictures of and TFAM in brain. (**H**) Quantified results of TFAM in brain. (**I**) Representative pictures of CMPK2 and TFAM in brain. (**J**) Quantified results of CMPK2 in brain. Data are expressed as the mean ± SD. ^#^ *p* < 0.05, ^##^ *p* < 0.01 vs. the Sham group and * *p* < 0.05, ** *p* < 0.01 vs. the MCAO group. Magnification: 100× and 200×.

Following ischemia, microglia are activated and accumulate in the ischemic penumbra and other pathological regions, thereby exacerbating the inflammatory response. Consequently, the degree of microglial activation around the ischemic penumbra reflects the severity of inflammation. Iba1 is commonly used to detect microglia. The quantitative IHC analysis results of microglial cell density in the ischemic penumbra are shown (Figure 3A–C). Compared to the sham-operated group, the MCAO group exhibited increased both the number and density of Iba-positive cells in the ischemic penumbra, extending even into the infarcted area; in contrast, the CSP group showed significantly reduced Iba-positive cell counts and densities. These findings suggest that CSP may modulate subsequent inflammatory responses by suppressing excessive microglial activation.

To investigate the effect of CSP on oxidative DNA damage in the brains of MCAO rats, we used IHC techniques. Compared with the Sham group, the area and percentage of 8-OHdG-positive cells in the ischemic penumbra were significantly increased. In contrast, the area and percentage of 8-OHdG positive cells in the CSP group were significantly reduced compared to the MCAO group (Figure 3D–F). These findings suggest that CSP attenuated oxidative DNA damage in the ischemic brain.

Ox-mtDNA generally stems from the direct attack of mtDNA by oxidative stress factors such as ROS. Under pathological conditions, reduced binding of TFAM to mtDNA impairs the structure of mitochondrial nucleoids, lowering mtDNA stability and increasing its exposure, which renders mtDNA more susceptible to oxidative damage. Thus, to investigate whether CSP affects mitochondrial DNA-associated homeostasis following ischemic injury, we measured the expression levels of CMPK2 and TFAM in brain tissues of MCAO rats. Compared with the Sham group, the MCAO group exhibited significantly increased CMPK2 and reduced TFAM protein expression, suggesting enhanced CMPK2-associated mitochondrial nucleotide metabolism and altered mtDNA maintenance under ischemic conditions. In contrast, CSP treatment significantly reduced CMPK2 and increased TFAM expression compared with the MCAO group, indicating that CSP can improve ischemic injury in rats by ameliorating mitochondrial dysfunction in microglia through CMPK2 (Figure 3G–J).

### 3.4. CSP Alleviated OGD-Induced Damage by Ameliorating Mitochondrial Dysfunction in Microglia Through CMPK2

**Figure 4:**
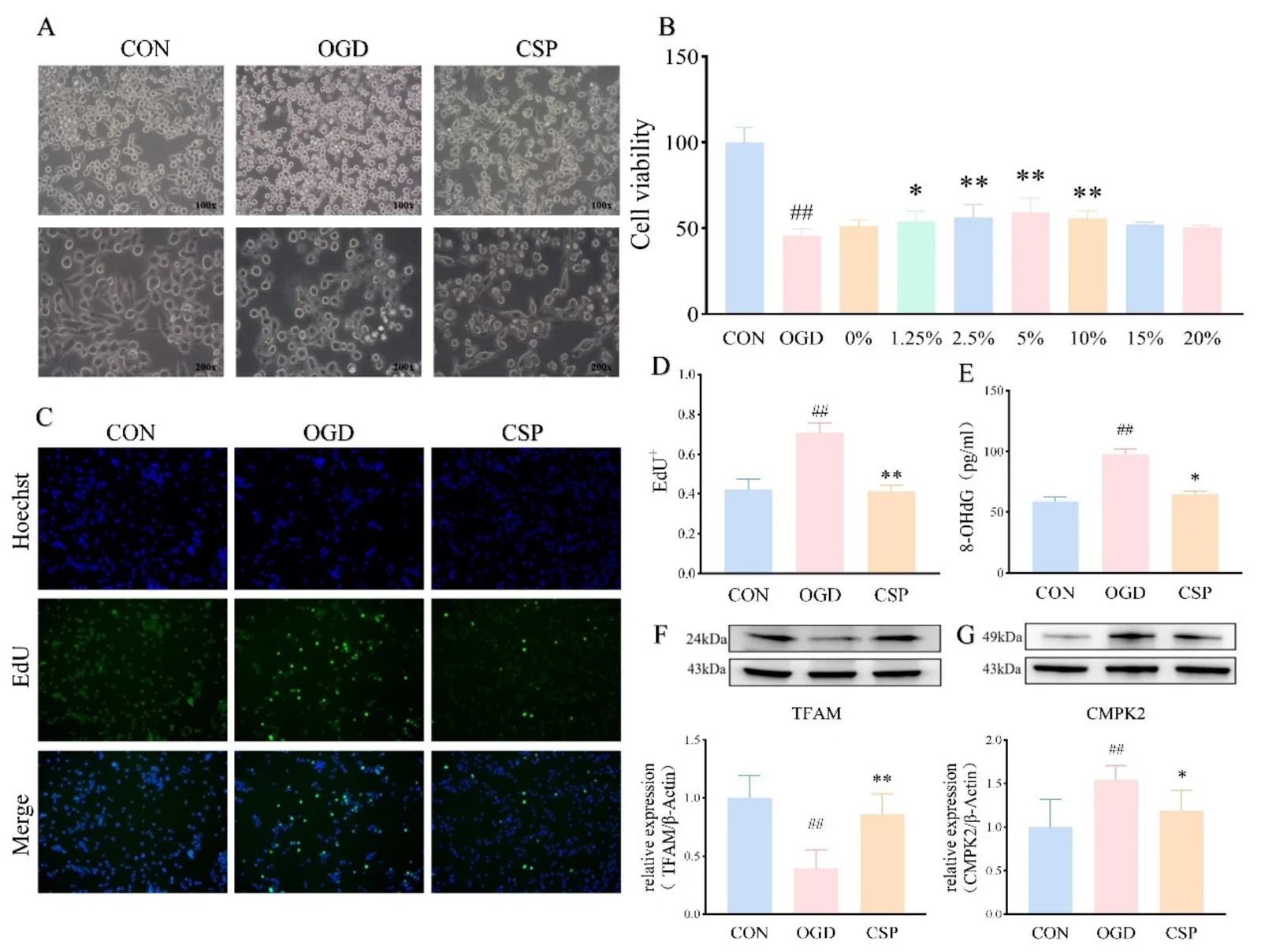
Microglial Activation, Oxidative Damage and Mitochondrial Protein Changes in Ischemic Penumbra of BV-2 cells. (**A**) Microscopic morphology of microglia BV-2 under light microscopy. (**B**) Survival rate of BV-2 cells under OGD exposure (*n* = 10). (**C**) Representative immunofluorescence images showing EdU incorporation and Hoechst-stained nuclei in BV-2 cells after OGD exposure (*n* = 6). (**D**) Quantification of EdU fluorescence intensity, with emphasis on extranuclear EdU signals. (**E**) 8-OHdG ELISA detection in BV-2 cells of each group (*n* = 6). (**F**) Representative Western blot images of TFAM and quantified results of TFAM in BV-2 cells (*n* = 6). (**G**) Representative Western blot images of CMPK2 and quantified results of TFAM in BV-2 cells (*n* = 6). Data are expressed as the mean ± SD. ^##^ *p* < 0.01 vs. the CON group and * *p* < 0.05, ** *p* < 0.01 vs. the OGD group. For in vitro figures, “CSP” denotes cells pretreated with CSP-containing serum before OGD induction.

Since microglial activation was markedly reduced by CSP in the ischemic penumbra, we next used OGD-exposed BV-2 microglial cells to further examine whether CSP modulates microglial injury and inflammatory signaling under ischemia-like conditions. Microglial cells exhibit high morphological plasticity. Following ischemic stroke, microglial cells undergo a series of changes to respond to various types of neural injury. We used an OGD model to simulate in vivo cerebral ischemia. Light microscopy results revealed that under normal conditions, BV-2 cells appeared round or spindle-shaped; after OGD exposure, BV-2 cells exhibited significant polarization with numerous floating dead cells; in the CSP group, BV-2 cells displayed synaptic shortening with minimal polarization (Figure 4A). CCK-8 assay results show OGD exposure reduced the survival rate of BV-2 cells, whereas addition of serum containing varying concentrations of CSP improved this phenomenon. The highest cell survival rate was observed at a 5% CSP concentration, which was selected as the condition for subsequent experiments (Figure 4B).

The increased EdU-positive signal in OGD-exposed BV-2 cells was predominantly distributed in the extranuclear region and showed limited overlap with DAPI-stained nuclei. This pattern suggests that OGD-induced EdU incorporation was unlikely to primarily reflect nuclear DNA synthesis (Figure 4C,D). Meanwhile, Western blot analysis showed that CMPK2 expression was significantly increased, whereas TFAM expression was markedly decreased in OGD-exposed BV-2 cells compared with the CON group. In contrast, CSP treatment significantly reduced CMPK2 expression and restored TFAM expression compared with the OGD group (Figure 4F,G). These results suggest that CSP may attenuate OGD-induced alterations in CMPK2/TFAM expression and DNA synthesis-associated changes in BV-2 cells. Similarly with in vivo experiment, we investigated the effects of CSP on oxidative DNA damage in BV-2 microglial cells using ELISA analysis. Compared with the CON group, the level of 8-OHdG, a marker of oxidative DNA damage, was significantly increased in the OGD group, whereas CSP treatment markedly reduced the 8-OHdG level compared with the OGD group (Figure 4E).

### 3.5. CSP Alleviated Cerebral Ischemic Injury in Rats by Inhibiting NLRP3 Inflammasome-Mediated Pyroptosis

**Figure 5:**
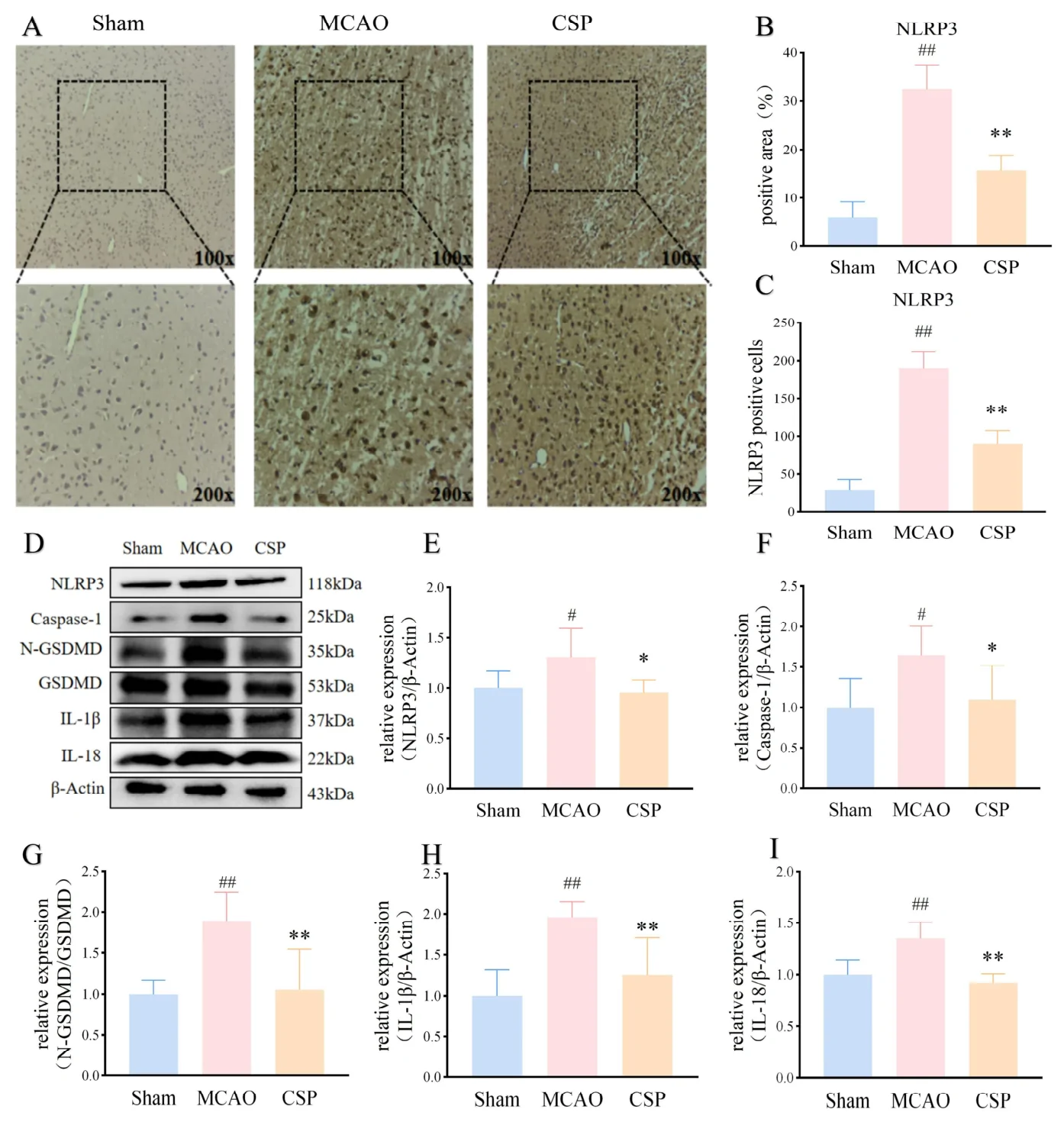
Expression of pyroptosis mediated by NLRP3 inflammasome in brain (*n* = 6). (**A**) IHC images demonstrate the expression of NLRP3 in the ischemic penumbra. (**B**) Calculation of NLRP3 positive area. (**C**) Quantitative diagram of NLRP3 positive cells. (**D**) Representative pictures of pyroptosis-related and inflammatory proteins in brain. (**E**) Quantified results of NLRP3, Caspase-1 (**F**), *N*-GSDMD (**G**), IL-1β (**H**) and IL-18 (**I**) in brain. Data are expressed as the mean ± SD. ^#^ *p* < 0.05, ^##^ *p* < 0.01 vs. the Sham group and * *p* < 0.05, ** *p* < 0.01 vs. the MCAO group. Magnification: 100× and 200×.

To determine whether CSP can inhibit NLRP3-related pyroptosis signaling, we examined the IHC expression of NLRP3 and the WB expression of NLRP3, Caspase-1, *N*-GSDMD, IL-1β, and IL-18 in brain tissue. Compared with the Sham group, the area and percentage of NLRP3 positive cells were significantly increased. Similarly, the area and percentage of NLRP3 positive cells in the CSP group were significantly reduced compared to the MCAO group (Figure 5A–C). Compared with the Sham group, the MCAO group exhibited significantly elevated levels of pyroptosis related proteins NLRP3, Caspase-1 and *N*-GSDMD, and inflammatory factors IL-1β and IL-18. In contrast to the MCAO group, the CSP group showed significantly reduced levels of NLRP3, Caspase-1, *N*-GSDMD, IL-1β and IL-18. These results indicated that CSP may alleviate cerebral ischemic injury in rats by inhibiting NLRP3 inflammasome-mediated pyroptosis (Figure 5D–I).

### 3.6. CSP Alleviated OGD-Induced Damage in BV-2 Cells by Inhibiting NLRP3 Inflammasome-Mediated Pyroptosis

**Figure 6:**
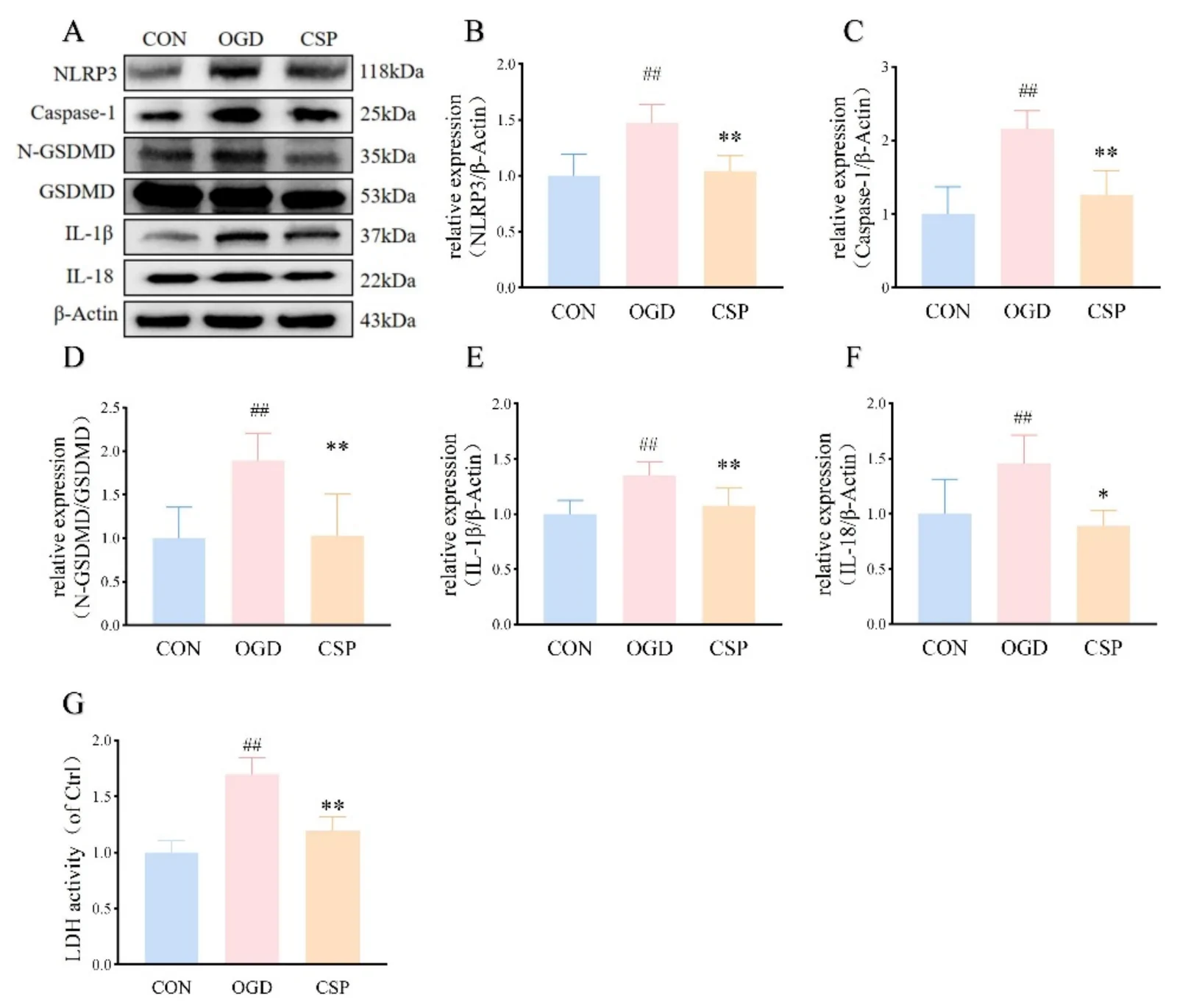
Expression of pyroptosis mediated by NLRP3 inflammasome in BV-2 cells (*n* = 6). (**A**) Representative pictures of pyroptosis-related and inflammatory proteins in BV-2 cell. Quantified results of NLRP3 (**B**), Caspase-1 (**C**), *N*-GSDMD (**D**), IL-1β (**E**), IL-18 (**F**) in BV-2 cells. (**G**) LDH release in BV-2 cells. Data are expressed as the mean ± SD. ^##^ *p* < 0.01 vs. the CON group and * *p* < 0.05, ** *p* < 0.01 vs. the OGD group.

Consistent with the in vivo findings, we performed quantitative protein analysis of NLRP3, Caspase-1, *N*-GSDMD, IL-1β, and IL-18 in BV-2 cells. Compared with the CON group, the OGD group exhibited significantly increased levels of the pyroptosis-related proteins NLRP3, Caspase-1 and *N*-GSDMD, as well as the inflammatory cytokines IL-1β and IL-18. In contrast, CSP treatment significantly reduced these changes (Figure 6A–F). LDH release in the culture supernatant was also significantly increased after OGD exposure and decreased following CSP treatment, indicating that CSP attenuated pyroptosis-related cell injury in BV-2 cells (Figure 6G).

## 4. Discussion

In our preliminary study, pre-administration of Cordyceps sinensis has been demonstrated to exert protective effects in both in vitro and in vivo models of ischemic stroke [28]. Although natural Cordyceps sinensis has significant medicinal value, its scarcity in the wild and high cost severely limit its clinical application. Advances in artificial cultivation technologies have enabled the large-scale extraction of bioactive components from Cordyceps sinensis. As one of its most abundant bioactive macromolecular components, CSP can be extracted at scale, thereby facilitating standardized preclinical investigation and future translational research. CSP has been reported to possess multiple pharmacological effects including anti-inflammatory, antioxidant, and immunomodulatory properties. In the present study, NXT was included as a positive control because it has been widely used in China for ischemic stroke management and has been reported to reduce infarct injury and improve neurological function in MCAO rats [8]. In clinical practice, NXT has been widely used in China for the management of patients with IS for many years [29,30]. The experimental results demonstrate that CSP intervention significantly reduces the infarct volume in MCAO rats, restores cerebral blood flow, attenuates cerebral edema severity, improves neurological function scores, and ameliorates histopathological changes in brain tissue, supporting its neuroprotective potential in the MCAO model. Additionally, we observed that CSP produced protective effects in the same direction as NXT, suggesting its potential clinical translational value.

The morphological dynamic changes of microglia reflect their functional state [31]. Prolonged exposure to hyperactive (amoeboid) microglia leads to irreversible neuronal loss [32]. This experimental study revealed that microglia in rat brain tissue rapidly activated and aggregated in the ischemic penumbra following MCAO modeling. After OGD exposure, BV-2 cells exhibited shrinkage and synaptic shortening. Under CSP intervention, both the Iba1-positive cells in the brains of MCAO rats and the BV-2 cells under OGD conditions exhibited improvements in both morphology and quantity. Therefore, we conclude that CSP maintains homeostasis of microglia in MCAO-modelled rat brain tissue and of BV-2 cells post-OGD exposure.

Recent studies have revealed that mitochondrial dysfunction is involved in multiple pathological processes of IS, playing a pivotal role in energy metabolism imbalance, oxidative stress [15], and neuroinflammation [16]. Mitochondria serve as a major source of reactive oxygen species (ROS) and have been demonstrated to amplify inflammatory responses through pathways such as mitochondrial ROS (mtROS) [33]. Given the close relationship between mitochondrial dysfunction, oxidative stress, and neuroinflammation, and considering the anti-inflammatory and antioxidant properties of CSP, we hypothesized that CSP might alleviate ischemic brain injury, potentially through modulation of mitochondrial function. As the central hub of cellular energy metabolism, newly synthesized mtDNA may undergo oxidative modification, forming oxidized mtDNA (ox-mtDNA), inducing intense inflammatory responses. Specifically, following MCAO or OGD modeling, the expression of CMPK2 increases and localizes to mitochondria. Initially identified as an interferon-stimulated gene (ISG) playing a critical role in antiviral immunity, recent studies have demonstrated CMPK2 involvement in the pathogenesis of various diseases, including atherosclerosis [34], cerebral calcification [35], systemic lupus erythematosus [36], and Zika virus infection [37], with its role in inflammatory disease progression increasingly elucidated. Research by Guan et al. demonstrated that CMPK2 in microglia promotes neuroinflammation and brain injury following ischemic stroke [38]. CMPK2 is the rate-limiting enzyme for inflammatory-associated novel mitochondrial DNA synthesis. It preferentially phosphorylates pyrimidine deoxyribonucleotide monophosphates (such as dCMP and dUMP) into their corresponding diphosphate forms, dCDP and dUDP, thereby enhancing the availability of mitochondrial dNTPs required for the synthesis of new mtDNA [39]. Under normal physiological conditions, mitochondrial transcription factor A (TFAM) packages mtDNA into a compact structure to protect it from oxidative damage [40]. However, ischemia-induced neuronal death triggers excessive activation and aggregation of microglia in the ischemic penumbra [11], accompanied by substantial production of mtROS within mitochondria [41]. Reduced TFAM expression may impair the proper packaging of newly synthesized mtDNA, leaving it in a more exposed and unstable state that is potentially more vulnerable to mtROS-mediated oxidation [42]. The experimental findings of this study are consistent with this hypothesis. In both MCAO rats and OGD-exposed BV-2 cells, CMPK2 expression was significantly increased, whereas TFAM expression was reduced, suggesting ischemia-induced disturbance of mitochondrial DNA-associated homeostasis. Notably, EdU staining in OGD-exposed BV-2 cells showed increased extranuclear EdU-positive signals with limited overlap with DAPI-stained nuclei, indicating that OGD-induced DNA synthesis-related changes were unlikely to primarily reflect nuclear DNA replication. Together with CMPK2 upregulation and TFAM downregulation, this finding supports the possibility that OGD may enhance mtDNA-associated DNA synthesis while reducing TFAM-mediated mtDNA maintenance. Conversely, CSP treatment decreased CMPK2 expression, restored TFAM expression, and reduced oxidative DNA damage. Based on these findings, we speculate that CSP may attenuate ischemia-induced mitochondrial DNA-related stress by suppressing CMPK2-associated DNA synthesis-related changes and improving TFAM-dependent mtDNA stability, thereby reducing DNA oxidative damage and subsequent inflammatory signaling.

It is noteworthy that newly synthesized or oxidized mtDNA may be released into the cytosol through mitochondrial membrane permeabilization pathways involving mPTP or VDAC1 [20]. These unstable mtDNA molecules are readily oxidized by ROS into ox-mtDNA, whether they remain inside mitochondria or enter the cytosol. Within mitochondria, large fragments of ox-mtDNA are cleaved by FEN1 into smaller pieces, which can then pass through the mPTP into the cytosol, thereby exacerbating tissue damage. Because oxidized DNA products may redistribute beyond mitochondria under stress conditions, and because mPTP opening may facilitate mtDNA release, we quantified 8-OHdG at the whole-cell level. However, this measurement reflects total cellular DNA oxidation and does not specifically distinguish mtDNA from nuclear DNA damage. The results showed a marked increase in 8-OHdG expression after MCAO, whereas the CSP group exhibited a significant reduction. Therefore, our data suggest that CSP attenuates overall cellular DNA oxidative damage after ischemic injury. Nevertheless, because 8-OHdG was measured in whole-cell lysates, further experiments are required to determine whether this protective effect is specifically attributable to reduced mtDNA oxidation.

Given that oxidized mitochondrial DNA (ox-mtDNA) released into the cytoplasm is an important endogenous damage-associated molecular pattern (DAMP), it has been widely reported to promote NLRP3 inflammasome activation and facilitate inflammatory signaling [43]. Therefore, the cytoplasmic accumulation of ox-mtDNA is considered a critical link between mitochondrial oxidative damage and subsequent neuroinflammatory responses. Our experimental results demonstrated that CSP effectively suppresses the upregulated expression of NLRP3 in brain tissues of MCAO-model rats and BV-2 cells following OGD exposure. The assembly of NLRP3 inflammasome activates Caspase-1, which cleaves precursor IL-1β and IL-18 into mature forms [44]. Importantly, activated Caspase-1 cleaves GSDMD to produce *N*-GSDMD, forming pores approximately 10–20 nm in diameter on the cell membrane, allowing the release of mature inflammatory factors such as IL-1β and IL-18 into the extracellular space, leading to secondary inflammatory damage [45]. Concurrently, water and ions from the extracellular space enter through these pores, resulting in osmotic swelling and cell deformation, ultimately causing complete plasma membrane rupture and release of cellular contents (e.g., LDH and HMGB1) [46]. To verify whether CSP can inhibit the NLRP3-mediated pyroptosis pathway, we detected the expression levels of cleaved Caspase-1, *N*-GSDMD, IL-1β, and IL-18 in MCAO or OGD models. The results showed that CSP inhibited the upregulation of their expression in the brain tissue of MCAO rats. Consistent results were obtained when detecting microglia BV-2 cells after OGD exposure. In addition, CSP reduced LDH release after OGD exposure, which is consistent with attenuation of membrane damage and supports the involvement of pyroptotic cell death.

Overall, the present study demonstrates that CSP exerts pronounced neuroprotective and anti-inflammatory effects in experimental ischemic stroke. Both in vivo and in vitro results consistently showed that CSP effectively attenuated ischemic injury, reduced inflammatory responses, and suppressed the expression of key pyroptosis-related molecules. Importantly, these protective effects are more appropriately attributed to specific regulation of post-ischemic inflammatory signaling and neuronal injury, with potential involvement of neurovascular protective pathways. Mechanistically, the protective effects of CSP may be closely associated with the regulation of mitochondrial dysfunction-related inflammatory signaling, particularly through downregulation of CMPK2, improvement of TFAM-associated mitochondrial DNA maintenance, potential attenuation of oxidized mtDNA-related inflammatory signaling, and subsequent inhibition of NLRP3 inflammasome-mediated pyroptosis. These findings provide further insight into the pharmacological basis of CSP and support its potential value for further evaluation in models of ischemic stroke. Nevertheless, the present results are preclinical and do not establish efficacy in humans. Further evaluation of whether CSP is suitable for preventive use, particularly in populations at increased risk of ischemic stroke, with attention to safety, dose selection, and preliminary efficacy may be a feasible strategy.

An important unresolved issue is how orally administered CSP may affect pathological processes in the ischemic brain. As a polysaccharide preparation, CSP is unlikely to readily cross an intact blood–brain barrier, and there is currently no direct evidence that CSP enters the brain parenchyma after oral administration. Therefore, the present findings should not be interpreted as the proof that CSP directly reaches neurons, microglia, or other brain parenchymal cells in vivo. Similarly, in vitro experiments using serum from CSP-treated animals do not demonstrate that CSP could cross the blood–brain barrier. Rather, these findings suggest that serum obtained after oral CSP administration contains bioactive components, metabolites, or secondary mediators that can modulate BV-2 cell responses when directly applied to the cells. It is more likely to reflect the bioactive ingredients or metabolites produced after CSP administration, which can regulate the response of BV-2 cells when directly applied to the cells.

However, there are still several limitations in this study. First, although CSP attenuated MCAO/OGD-induced NLRP3 inflammasome activation, pyroptosis, CMPK2 upregulation, and TFAM downregulation, the current evidence is mainly correlative and does not prove that CSP directly targets the CMPK2/TFAM pathway. Further gene knockdown, overexpression, or pathway inhibition studies are needed to confirm the causal mechanism. Second, the evidence for mtDNA damage and release remains indirect. Although 8-OHdG, EdU incorporation, and related protein changes were assessed, mtDNA synthesis, oxidative mtDNA damage, cytosolic mtDNA release, and its interaction with the NLRP3 inflammasome were not directly examined. Therefore, the role of oxidized mtDNA should be considered a plausible hypothesis rather than a directly demonstrated mechanism. Third, some in vivo mechanistic analyses were performed using whole brain tissue rather than isolated microglia; therefore, contributions from other brain cell types cannot be excluded. Future studies should evaluate CSP effects on antioxidant capacity, ROS generation, mtDNA release, and inflammasome assembly using cell-specific approaches, and should further clarify whether modulation of mitochondrial metabolism and CMPK2-related signaling is a key mechanism underlying its neuroprotective effects in ischemic stroke models.

## 5. Conclusions

In conclusion, CSP treatment was associated with neuroprotective and anti-inflammatory effects in experimental ischemic stroke. In our study, CSP intervention was accompanied by decreased CMPK2 expression and increased TFAM expression, suggesting a potential improvement in mitochondrial DNA maintenance. These changes may contribute to reduced oxidized mtDNA-related inflammatory signaling and attenuation of NLRP3 inflammasome-mediated pyroptosis. Overall, these findings suggest that CSP may have therapeutic potential for ischemic stroke, although further studies are needed to clarify the underlying mechanisms.

## Data Availability

The data supporting the findings of this study are available from the corresponding author upon reasonable request.

## Supplementary Materials

The following supporting information can be downloaded at https://www.mdpi.com/article/10.3390/nu18162591/s1. Table S1. Modified neurological severity scores (mNSS).
