# Supplementary material for: Cordyceps Polysaccharides Attenuate Post-Ischemic Inflammatory Damage in a Rat Stroke Model with Associated Modulation of the mtDNA/NLRP3 Inflammasome-Related Pyroptosis Pathway
Source: Nutrients. 2026 Aug 7;18(16):2591. doi: 10.3390/nu18162591 (PMC13517037; doi:10.3390/nu18162591)
Supplement: Supplementary file 1 [file nutrients-18-02591-s001.zip › nutrients-4425168-supplementary.pdf]

Table S1. Modified neurological severity scores (mNSS).[1]

Items	Points
**Motor tests**	**6**
Hanging rat by tail (0–3)	3
Forelimb flexion	1
Hindlimb flexion	1
Head moved >10°to vertical axis within 30s	1
Placing rat on the floor (0–3)	3
Normal walk	0
Inability to walk straight	1
Proprioceptive test (deep sensory)	2
Fall down to the paretic side	3
**Sensory tests**	**2**
Visual and tactile placing	1
Proprioceptive test (deep sensory)	1
**Beam balance tests**	**6**
Grasps side of beam	1
Hugs the beam and one limb falls down from the beam	2
Hugs the beam and two limbs fall down from the beam, or spin on beam (> 60 s)	3
Attempt to balance on the beam but falls off (> 40 s)	4
Attempt to balance on the beam but falls off (> 20 s)	5
Falls off with no attempt to balance or hang onto the beam	6
**Reflexes (blunt or sharp stimulation) absent of:**	**4**
Pinna reflex (head shake after touching the auditory meatus)	1
Corneal reflex (eye blinking after touching the cornea with a cotton)	1
Startle reflex (motor response to a brief noise)	1
Seizures, myoclonus, myodystonia	1
**Maximum points**	**18**

Score: 13-18 severe; 7-12 moderate; 1-6 mild injury.

[1] Chen, J. *et al.* Intravenous Administration of Human Umbilical Cord Blood Reduces Behavioral Deficits After Stroke in Rats. *Stroke* **32**, 2682–2688 (2001).
